# The Slowest Shared Resonance: A Review of Electromagnetic Field Oscillations Between Central and Peripheral Nervous Systems

**DOI:** 10.3389/fnhum.2021.796455

**Published:** 2022-02-16

**Authors:** Asa Young, Tam Hunt, Marissa Ericson

**Affiliations:** ^1^Department of Psychological and Brain Sciences, University of California, Santa Barbara, Santa Barbara, CA, United States; ^2^Department of Psychology, University of Southern California, Los Angeles, CA, United States

**Keywords:** resonance, interoception, consciousness, EEG, embodied cognition, coupled oscillators

## Abstract

Electromagnetic field oscillations produced by the brain are increasingly being viewed as causal drivers of consciousness. Recent research has highlighted the importance of the body’s various endogenous rhythms in organizing these brain-generated fields through various types of entrainment. We expand this approach by examining evidence of extracerebral shared oscillations between the brain and other parts of the body, in both humans and animals. We then examine the degree to which these data support one of General Resonance Theory’s (GRT) principles: the Slowest Shared Resonance (SSR) principle, which states that the combination of micro- to macro-consciousness in coupled field systems is a function of the slowest common denominator frequency or resonance. This principle may be utilized to develop a spatiotemporal hierarchy of brain-body shared resonance systems. It is predicted that a system’s SSR decreases with distance between the brain and various resonating structures in the body. The various resonance relationships examined, including between the brain and gastric neurons, brain and sensory organs, and brain and spinal cord, generally match the predicted SSR relationships, empirically supporting this principle of GRT.

## Introduction

A nested hierarchy of electromagnetic (EM) fields spans the entire human physiology, encompassing the cortex, deep brain structures, and an extracerebral network throughout the body (Hales, 2017; Klimesch, 2018). This system of EM fields appears to synchronize, in various ways and to varying degrees, neural activity in peripheral neural clusters, such as the stomach and heart, to the brain’s rhythms, maintaining a generally steady state, during waking consciousness, of “electromagnetic homeostasis” (De Ninno and Pregnolato, 2017). All of this EM field activity supervenes upon a neuroanatomical backbone that is as stable as the fields are dynamic.

This review summarizes the current research on shared electromagnetic field resonance among (1) the human brain and its peripheral nervous system; (2) similar activity observed in other animals; and (3) the implications of these interactions for electromagnetic field theories of consciousness, specifically the General Resonance Theory (GRT) of consciousness (Hunt, 2011, 2020; Hunt and Schooler, 2019). GRT postulates that all matter resonates and has some iota of associated consciousness. Shared resonance is achieved when matter resonates in proximity with other matter at the same frequency, or harmonics thereof, over a sufficient duration, resulting in the combination of micro-consciousnesses into larger, more complex macro-consciousness. GRT is a proposed solution to the Combination Problem (Hunt and Schooler, 2019), which is often leveled as a critique of panpsychist approaches to consciousness. A key motivation of GRT is the transformation of panpsychism from a philosophical position into an empirical theory of consciousness.

EM field theories of consciousness, of which GRT is one type, often posit that neuronally generated EM fields (Buzsáki et al., 2012; Hales, 2017; Chiang et al., 2019) are the primary seat of consciousness. GRT suggests a shared resonance between various nested EM fields and the specific kinds of information processing made possible with resonating EM fields is necessary and sufficient for mammalian consciousness. It differs in this respect from Integrated Information Theory (Oizumi et al., 2014), a widely discussed theory of consciousness, that suggests information processing and its integration are necessary and sufficient for consciousness. We can measure the dynamics of these EM fields by, for example, distinguishing five primary EEG frequency bands: delta (0.2–3 Hz), theta (3–8 Hz), alpha (8–12 Hz), beta (12–27 Hz), and gamma (27–100 Hz) (Fields, 2020), and then measuring their strength and interactions throughout the brain. It is not arbitrary that there are five main bands because recent data supports the view that the mammalian brain often achieves, particularly during times of high performance, a binary (harmonic) hierarchical relationship between each band (Klimesch, 2018; Rassi et al., 2019; Rodriguez-Larios et al., 2020). For example, the middle range of theta is twice that of delta, and also for alpha in relation to theta, etc. This binary hierarchy supports the notion that the EM fields generated by the brain are indeed causal rather than epiphenomenal (Klimesch, 2018; Rassi et al., 2019). The heart-brain, gastric-brain, and retinal-brain shared resonance relationships surveyed in the current paper appear to, in some manner, entrain cerebral EM fields, and vice versa (all physical reactions are necessarily bi-directional).

“Consciousness” will be defined here for clarity as the “what it is to be like” (subjective feeling) of the EM field system produced by the brain and peripheral nervous system (Hales et al., 2021, in progress). While Nagel (1974) asked “What is it like to be a bat,” we reframe this as: “What is it like to be the EM field system produced by the brain of that bat?”

Theories of embodied cognition propose an inherent capacity of the body to constrain, regulate, and distribute cognitive processes across the organism (Thompson and Varela, 2001; Fuchs, 2009; Foglia and Wilson, 2013). We follow this tradition by suggesting that cognitive processes and endogenous rhythms are tightly coupled and are the pathways through which the body regulates an agent’s cognitive activity over both space and time. The structuring of the neuronally generated cerebral fields by the oscillatory activity of distant neural clusters is one such manifestation of this interconnected network of embodied cognition. Similar theories argue that the experienced “self” emerges from the constant stream of afferent signals the brain receives from the various bodily organs (Park and Tallon-Baudry, 2014).

The alignment of EM field oscillations, or oscillatory integration windows, between the brain and other organs seems to be a key part of the conditions that allow a unified consciousness to emerge (Strogatz, 2012; Riddle et al., 2021, in progress). Gupta et al. (2016) provides evidence of preferred windows of oscillation cycles in locust Kenyon cells that more effectively integrate information and separate signal from noise. We also review various brain-body shared resonances in rats and octopi, and these data provides at least preliminary evidence that extracerebral shared resonance is not solely a human or mammalian phenomenon.

In fact, if we follow in the embodied cognition tradition, we hypothesize that the embodied mind, represented here by various brain-body shared resonances in organizing a unified organismic consciousness, must have been present in terrestrial organisms as early as the Cambrian explosion (Trestman, 2013), and probably far earlier. This suggests that embodied cognition may have emerged at roughly the same time as the ancestral bilaterian, the first animal with an organization for left and right, top and bottom, front and back (Godfrey-Smith, 2013). Embodied cognition and brain-body coupling is likely present in many, if not all, modern organisms, from the highly centralized nervous systems of *Homo sapiens* to the highly decentralized, split-brain-esque nervous system of the *Octopus vulgaris* (Godfrey-Smith, 2020).

Our analysis seeks to shed light on GRT’s SSR principle: that the shared resonance frequency between neural clusters decreases with the spatial distance separating them (Hunt, 2020). The slowest common denominator frequency, or Slowest Shared Resonance (SSR), accordingly, defines the boundaries of each conscious entity in each moment. The inverse relationship between frequency of the SSR and distance between neural clusters is due to the physics of wave propagation. Lower frequencies travel faster (Dehaene, 2014), thus information to the brain will be carried upon increasingly lower frequency signals as the distance between the two clusters increases.

Cross-frequency coupling (CFC), the modulation of a faster frequency by a slower rhythm through harmonics or phase-amplitude coupling, is a mechanism by which functional systems may be integrated across varying spatiotemporal scales (Canolty and Knight, 2010) and a necessary component of the spatiotemporal relations underlying the combination of consciousness that is suggested by GRT (for a review of the relationship between disordered states of consciousness and altered cross-frequency couplings as supportive of the causality conferred to CFC, see Cai et al., 2020).

The evidence reviewed is largely supportive of GRT’s SSR prediction. Further research is needed, however, both in improving our understanding of the various spatial and temporal causal hierarchies present, as well as for its application in clinical settings, if we are to succeed in our effort to make GRT a mature and empirically founded theory of consciousness.

## The Oscillatory Hierarchy of Brain and Body Rhythms

The trend toward considering EM field oscillations as causal rather than epiphenomenal has been gradual, as new evidence has come to light and new paradigms developed. Here, we will review several recent studies that speak to the causal role of cerebral fields in cognition and consciousness.

One such method of testing the cognitive role of certain neural oscillations has been through the application of artificial oscillatory stimulation using rhythmic transcranial magnetic stimulation (rTMS), transcranial alternating current stimulation (tACS), and transcranial direct current stimulation (tDCS)—collectively termed transcranial brain stimulation (TBS).

Klimesch et al. (2003) applied rTMS to subjects’ frontal and parietal regions (specifically P6 and Fz cortical sites) at their individual alpha frequencies which was found to increase cognitive performance in mental rotation tasks by influencing the dynamics of their alpha desynchronization. Similarly, Wilsch et al. (2018) fortified cortical entrainment to the speech envelope at 3–8 Hz (Fujii and Wan, 2014), successfully modulating sentence comprehension. In a broad review of neurostimulation studies, Thut et al. (2011) supports the efficacy of the modality in modulating cognitive behaviors through the alteration of cortical oscillatory networks. It’s application on the clinical end has found substantial success directly treating the neural networks associated with disordered consciousness in Alzheimer’s disease (Nardone et al., 2015; Chang et al., 2018), anxiety (Kar and Sarkar, 2016), post-traumatic stress disorder (PTSD), and obsessive-compulsive disorder (Freire et al., 2020) to name a few among many psychopathologies.

Other empirical methods include observing the spontaneous, yet organized, coupling and decoupling of various brain waves during cognitive tasks. Rodriguez-Larios et al. (2020) recorded the enhanced transient occurrence of 2:1 (binary) harmonic cross-frequency coupling between alpha and theta when subjects engaged in effortful cognition. The same study also recorded a decrease (as compared to resting state) in alpha-theta coupling during a “mind emptiness” meditation task, an experimental condition opposite that of effortful cognition.

Samaha and Postle (2015) utilized flicker-fusion rate as a measure of visual experience in order to investigate the relationship between cortical alpha frequencies and temporal resolution. Subject’s individual alpha frequency predicted temporal resolution of visual perception (Samaha and Postle, 2015). A slower alpha frequency would process both flashes in a single oscillatory integration window with which the subject would report a fusion of the two flashes. A faster alpha frequency showed the two flashes were processed in different oscillatory windows and the subject reported the visual experience of two flashes. These results were taken as evidence that the alpha wave underlies the temporal granularity of visual perception, defining what was presented to the subject’s consciousness in each moment.

Başar (2008) describes the body as a unified network of oscillatory activity between the brain, spinal cord, and peristaltic organs—a system whose foundation is built on a frequency band hierarchy. The hierarchy proposed contained three tiers organized by frequency in descending order: the high frequency band (above 40 Hz), EEG frequencies, and the ultra-slow oscillations. Integrating results from Ruskin et al. (1999) and Allers et al. (2000, 2002), Başar (2008) hypothesized that the ultra-slow oscillations (0.001–1 Hz) originating from various elements of the autonomic nervous system (ANS) have the potential to organize the propagation and synchronization of neural oscillations in higher frequency bands. ANS feedback signals to the brain carry the input for bodily experience, contributing to the feelings of energy, fatigue, and relaxation (Başar, 2008). Barman and Gebber (1993) and Barman et al. (1995) recordings of sympathetic nerves in decerebrated cats demonstrated 10 Hz discharges between both subcortical structures and cardiac neurons. Başar (2008) concludes that these data support the presence of oscillatory links between the brain, spinal cord, and organs of the body.

**FIGURE 1:**
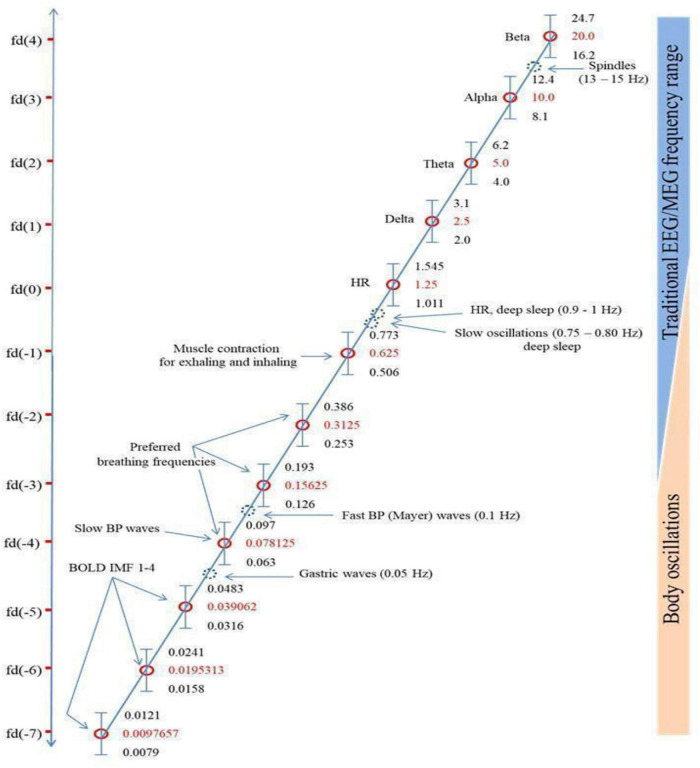
Klimesch’s (2018) figure illustrating the Binary Hierarchy Brain Body Oscillation Theory frequency architecture. Frequency bands, calculated according to the “golden mean rule” (see Klimesch, 2018), are depicted as vertical bars (bandwidths relative to the *y*-axis are not to scale). Frequencies lying outside the predicted bands are represented as dashed blue circles and are considered falling outside the binary hierarchy.

Klimesch’s (2018) “binary hierarchy brain body oscillation theory” suggests that the frequency of body oscillations can be predicted from brain oscillations, and vice versa, and that these different frequency domains fall on a predictable pattern across 11 or more “frequency domains.” He suggests that brain-body coupling is governed by either harmonic phase-to-phase coupling or phase-to-envelope coupling. The six endogenous rhythms that Klimesch focuses on are presented in Figure 1, which reproduces Figure 5 from Klimesch (2018), in descending order of frequency: (1) brain rhythms; (2) heart rate; (3) breathing frequency (BF); (4) blood pressure (BP); (5) gastric neuron basal rhythms; and (6) blood oxygen level dependent (BOLD measured with fMRI) signal. These rhythms generally remain coupled to brain wave frequency bands during waking states but become decoupled in sleep, although the body rhythms remain coupled to each other in binary integer ratios even in sleep (Rassi et al., 2019).

Another pattern not included sufficiently in Başar’s and Klimesch’s work is a shared resonance between cerebral EM fields, represented by interactions between the five brain wave frequency bands, and the various organs of the body, including gastric, retinal, and cardiac neurons (Klimesch does discuss gastric/cerebral resonance, as Figure 1 shows, but not the other resonance relationships we discuss below). We discuss four such shared resonance relationships in laying the groundwork for additional research into brain-body oscillatory links that have not yet been explored sufficiently, but are predicted by both Başar’s (2008) globally coupled oscillators model and Klimesch’s (2018) binary hierarchy brain body oscillation theory.

### Gastric-Brain Shared Resonance

Measured with the electrogastrogram (EGG), the peristaltic organs, primarily the stomach, emit a central frequency of 0.05 Hz (about one cycle every 20 s), an ultra-slow oscillation (Başar, 2008). This oscillation, the gastric basal rhythm, is emitted by the interstitial cells of Cajal—specialized cells in the stomach’s wall (Takaki, 2003). Richter et al. (2017) reported a stomach-brain resonance relationship by which EEG frequencies were phase amplitude coupled (PAC) to the gastric basal rhythm. PAC evaluates changes in the amplitude of a high frequency oscillation according to the phase of a lower frequency oscillation—a relationship that usually presents between distant neural clusters (Richter et al., 2017). This study provided evidence for a directional relationship between the 0.05 Hz ultra-slow oscillations of the stomach and the 8–12 Hz oscillations of the brain’s alpha waves, suggestive of a framework by which slow organ oscillations can, in fact, couple to the brain’s higher frequency waves. Richter notes that the relationship is primarily ascending with the greatest information transfer from stomach to brain due to the intrinsic nature of the stomach’s emitted pulses.

Rebollo et al. (2018) expanded on Richter’s gastric-alpha work by establishing oscillatory links between the stomach-brain interaction and the BOLD signal in resting state networks (RSN). This relationship arises due to the following dynamics: (1) the stomach’s extensive connections to cortical structures through neuroanatomical pathways (primarily Vagus nerve) and EM field resonance; (2) the gastric basal rhythms falling within the BOLD signal’s range of fluctuations (0.0079–0.0483 Hz; Klimesch, 2018); and (3) the PAC of alpha-gastric basal rhythm (Richter et al., 2017). The network is represented by synchronized fluctuations in BOLD signal in brain structures known to be coupled to the gastric basal rhythm, extrapolated from Richter’s earlier work. Rebollo et al. (2018) “identifies BOLD regions that go faster when the stomach goes faster, and slower when the stomach goes slower.”

Rebollo et al.’s (2018) study revealed a novel RSN by which two endogenous rhythms, BOLD and gastric basal rhythms, are phase-amplitude-coupled, but only partially (up to ∼17 percent maximum) to the brain’s alpha waves. The relationship is argued to be bi-directional in establishing an interoceptive sense (Huizinga, 2017, per the entrainment of cortical oscillations in the insular cortex) and maintaining homeostasis, but Rebollo agrees with Richter that there is a significant ascending influence of the stomach on the brain. The vagus nerve (VN), a significant carrier of information between the brain and the GI tract, is composed of eighty percent afferent fibers and twenty percent efferent fibers (Bonaz et al., 2018). This division in the VN validates Rebollo and Richter’s proposition of a significant ascending influence but in no way rules out descending influence. This gastric-brain shared EM field resonance is defined by the ultra-slow oscillations of the 0.05 Hz gastric basal rhythm partially resonating with the brain’s alpha wave. This is the slowest of the shared resonance relationships examined here, and the stomach’s neural cluster is farthest, of the organs examined herein, from the brain.

### Cardiac-Brain Shared Resonance

The heart is implicated in embodied cognition frameworks, specifically in its influence on emotional experience. For example, fear signals are judged as more fearful when the stimulus appears during a heartbeat as opposed to between beats (Garfinkel and Critchley, 2016). In fact, the resting state heartbeat induces phase synchronization between cortical regions in the theta frequency (3–8 Hz) comprising a five-module network known as the heartbeat-induced network (Kim and Jeong, 2019). Increased synchronization correlated with more positive mood as well as an inverse relationship with negative mood.

McCraty et al. (2009) sought to uncover a coherent heart-brain system of interaction by correlating emotional states with psychophysiological changes, citing the heart as the most powerful generator of endogenous rhythms in the body. The study identified six psychophysiological states to measure cardiac-brain resonance: mental focus, psychophysiological incoherence, psychophysiological coherence, relaxation, extreme negative emotion, and emotional quiescence. The state of psychophysiological coherence was specifically noted as a shared resonance state between brain and heart oscillations (McCraty et al., 2009). This state, which entails the global coupling of body rhythms to cardiac oscillations, is hypothesized to be causal in increasing performance and overall well-being (or the converse through desynchronization or dysregulation).

The study demonstrates, unsurprisingly, a bidirectional relationship between the heart and the brain, wherein emotional states are reflected in the heart rate variability (HRV) and the cognitive processes that shape emotion are modulated by HRV. The 0.1 Hz (10 s cyles) central heart resonant EM field frequency (measured with EKG), represented by a distinct high amplitude peak in the HRV power spectrum, synchronizes with alpha (derived from Wölk and Velden, 1987, 1989; replicated by McCraty et al., 2009) and beta (per McCraty et al., 2009) bands in the brain in psychophysiological coherent states. These 0.1 Hz oscillations reappear in PTSD patients undergoing Somatic Experiencing (SE) trauma resolution therapy when a threat response is successfully re-negotiated and constricted energy is subsided (Whitehouse and Heller, 2008).

The cardiac-brain shared resonance is defined by the 0.1 Hz heart resonant frequency with shared resonance occurring between alpha and beta EEG bands. In developing the ongoing pattern in this paper, the SSR for cardiac-brain shared resonance occurs at a frequency twice as fast as the 0.05 Hz of the gastric basal rhythm underlying gastric-brain shared resonance, and supports the SSR prediction that SSR will increase inversely with distance between neural clusters.

### Retinal-Brain Shared Resonance

Several studies provide evidence for oscillatory links between the retina and brain (Leszczynski and Schroeder, 2019; Leszczynski et al., 2020). Saccadic eye movements are generated rhythmically at 3–4 Hz by the observer to place the fovea—the area of the retina containing the densest concentration of photoreceptors—on a target. Saccadic rhythms reset the alpha phase (8–12 Hz) in the occipital lobe and theta phase (4–7 Hz) in the medial temporal lobe (Leszczynski and Schroeder, 2019). The rhythmic nature of the saccadic phase reset generates an entrainment, the largely uni-directional coupling of one oscillator to another (Lakatos et al., 2019), of alpha and theta EEG bands.

The alpha-theta entrainment aligns cerebral oscillatory integration windows to the flow of the rhythmic visual input from the retina, an exchange termed active visual sensing (Leszczynski et al., 2020). Active sensing is defined as fovea relocation through saccadic eye movements and sampling of different bits of visual information many times a second. The entrainment of alpha and theta to saccade onset rhythms amplifies neuronal responses to the incoming visual stream, thus lowering perceptual thresholds (Leszczynski and Schroeder, 2019). This hypothesis about the mechanism for these connections was confirmed in a later study (Leszczynski et al., 2020). In this sense, just as gastric-brain shared resonance appears to underlie interoception and cardiac-brain shared resonance contributes to emotional experience, retinal-brain shared resonance enacts a functional relationship to engage in active sensing.

The 3–4 Hz saccadic rhythm serves as the SSR for this retinal-brain shared resonance relationship with the alpha and theta brain waves. As a neural cluster significantly closer to the brain than either the stomach or heart, its SSR is a significantly faster frequency than the two previously discussed shared resonance relationships.

### Corticospinal Shared Resonance

The spinal cord, an important part of the central nervous system, engages in large-scale coherent neuronal firing with the brain’s motor cortex during movement. Using reaction time tasks, Schoffelen et al. (2005) manipulated hazard rates of the go-cue peri-task to measure “readiness” and the corticospinal interaction that underpin it. The hazard rate of an event is the conditional probability of the event occurring if it has not yet occurred. Readiness was represented by the subject’s response times (Riehle et al., 1997; Trillenberg et al., 2000). Measuring the left motor cortex neuronal group directly with magnetoencephalography (MEG) and the corresponding spinal neuronal group indirectly through the right musculus extensor carpi radialis longus with electromyogram (EMG), Schoffelen recorded beta (12–27 Hz) and gamma (27–100 Hz) coherence between the spinal cord and motor cortex. The experimental paradigm was split into two conditions: UP- and DOWN-schedule, referring to the hazard rate of the go-cue. Corticospinal gamma band coherence increased with the hazard rate in UP-schedule trials and decreased in DOWN-schedule trials. Corticospinal coherence in the beta band was present through every trial regardless of schedule. Results demonstrated corticospinal coherence in the gamma band and tightly coupled readiness-to-respond. Gamma band coherence was increased when the subject expected a physical movement.

This corticospinal to brain shared resonance with beta and gamma bands appears to underlie mechanical motion (van Wijk et al., 2012). However, differing from the other shared resonance systems surveyed, the evidence is inconclusive in establishing an entraining rhythm by the spinal cord to ground the corticospinal shared resonance, as discussed above for gastric basal rhythm (0.05 Hz), heart resonant frequency (0.1 Hz), or retinal saccadic rhythm (3–4 Hz). Although the preliminary evidence is promising, further research is needed to define the SSR of this particular system and integrate corticospinal shared resonance into the GRT spatiotemporal hierarchy.

## Principles of Spatial Coupling

Research discussed above supports the presence of oscillatory links between the brain and the heart, stomach, retina, and spinal cord. Subsequent studies will be needed to expand the framework to include shared resonances between the brain and neural clusters in the genitals, lungs, olfactory centers, and any combination thereof.

The pattern predicted by GRT’s SSR principle—distance inversely affects shared resonance frequency bands—is generally supported by current findings (Table 1). The retina, as the most proximal neural cluster to the brain, emits the highest frequency SSR (3–4 Hz), in the form of a saccade and associated neural activity resonating with theta and alpha EEG bands (Leszczynski and Schroeder, 2019; Leszczynski et al., 2020). The heart’s primary EM field rhythm, the heart resonant frequency at 0.1 Hz, resonates with alpha and beta brain waves (McCraty et al., 2009). The stomach, the organ most distant from the brain (of the organs reviewed herein), emits the slowest frequency SSR at 0.05 Hz gastric basal rhythm, resonating with the brain’s alpha rhythms (Richter et al., 2017; Rebollo et al., 2018). While research is suggestive of corticospinal shared resonance (Schoffelen et al., 2005), the evidence is inconclusive in establishing an SSR frequency underlying the observed resonance. We also include preliminary data on olfactory center and brain shared resonance as a plausible addition to the developing hierarchy.

**TABLE 1:** Slowest Shared Resonance (SSR) between coupled neural clusters.

Coupled system	Slowest Shared Resonance (SSR)	Estimated distance**
Retinal-brain	3–4 Hz	5 cm
Olfactory-brain*	0.16–0.33 Hz	7–10 cm
Cardiac-brain	0.1 Hz	30 cm
Gastric-brain	0.05 Hz	50 cm
Corticospinal-brain	N/A	15–50 cm (wide range due to length of spinal cord)

*Tentative placement.

**Rough estimate provided in normal stature adult human, provided for illustration only. Additional research is required to provide better estimates.

Zelano et al. (2016) findings are suggestive of links between nasal respiration rates and delta, theta, and beta waves in the human piriform cortex (PC), and limbic-related brain areas. Intracranial EEG recordings paired with concomitant nasal respiration recordings indicated cortical entrainment to the 0.16–0.33 Hz rhythm of the human respiratory cycle. This BF is hypothesized to act as an electrical pacemaker synchronizing activity in the PC, amygdala, and hippocampus. These results determined this entrainment to be functional in modulating emotional experience and memory. Fearful faces are identified with faster reaction times when presented during nasal inspiratory phase, but not surprised faces. Recognition tasks in memory tests find enhanced retrieval of stimuli when prompted during nasal inspiratory phase versus expiration. These findings are in line with those of Klimesch (2018), such that BF appears to be one of the primary organizing endogenous rhythms. Corcoran et al. (2018) echoes this view, hypothesizing respiration-brain coupling to constitute a global rhythm that structures higher-frequency brain activity. However, what differs from the previously discussed shared resonances is that olfactory-brain shared resonance does not appear to have an entraining neural rhythm apart from the entraining physiological rhythm. Giving credence to Zelano’s findings, the 0.16–0.33 rhythm of human nasal respiration (the olfactory-brain SSR) would place the shared resonance appropriately above cardiac-brain and gastric-brain, and just below retinal-brain shared resonance properly fitting in our developing spatial hierarchy (Table 1).

## Entrainment and Shared Resonance

The internal resonance properties of the cerebral field system, as the brain is entrained by and responds to external stimuli, supports the notion that EM field resonance is causal with respect to the dynamics of consciousness (Başar et al., 1975). The entrainment of cortical oscillations through the application of rhythmic auditory (Chatrian et al., 1960) and visual (Adrian and Matthews, 1934) stimuli is a deeply researched and well-substantiated tool in clinical settings for treating disordered states of consciousness (Siever and Collura, 2017), as well as a common method of laboratory paradigms for correlating neural oscillations with conscious and cognitive behaviors (Young, 2021, in press). This section surveys empirical examples in which resonance between the central and peripheral neural clusters, specifically between the heart and the brain, is driven by external stimuli.

The conscious control that can be exercised over respiration makes it unique among the fundamental rhythms. Breathing at a rate of 6 breaths per minute (0.17 Hz) evokes synchrony among cardiac measures (Shaffer et al., 2014) and induces coherence in the 0.1 Hz oscillations of the heart resonant frequency (Schwerdtfeger et al., 2020). The range of “coherent” or “resonance” breathing is between 4.5 (0.22 Hz) and 6.5 (0.15 Hz) breaths per minute depending on the cardiovascular disposition of the individual but all values fall within BF ranges of Klimesch’s brain-body frequency architecture (Figure 1; Klimesch, 2018). This breathing mode, in evoking the heart resonant frequency, affects the physiological and psychological regulation of negative affect (Schwerdtfeger et al., 2020). This is one avenue through which an ascending influence of HRV may partially entrain the brain’s endogenous rhythms and regulate cognition.

The bidirectional nature of the cardiac-brain correspondence is exemplified by the downward influence the brain’s EM oscillations exercises over the heart in audiovisual entrainment (AVE) studies. As referenced above, auditory and visual stimuli, separately or in combination, reliably entrain cortical oscillations. Francesco et al. (2013) administered alpha frequency AVE and, in doing so, increased HRV, a sign of positive cardiac health (van Ravenswaaij-Arts et al., 1993). McConnell et al. (2014), in a similar study, administered theta frequency binaural beats to individuals post-exercise. The measures of HRV recorded no significant change but the AVE-stimulated group reported greater feelings of relaxation. An acute influence over low and high frequency components of HRV were concluded. We may speculate that the greater success of Francesco et al.’s (2013) stimulation was due to the targeted frequency. Alpha, but not theta, resonates with the HRV signal (McCraty et al., 2009) and the manipulation of one end of the correspondence will alter the other.

## Shared Resonance in Other Animals

Cerebral and extracerebral EM field oscillations, as reviewed above, are not a phenomenon specific to humans. The frequency hierarchy we inherited from our distant evolutionary ancestors is, in many cases, very similar to that which humans and other mammals possess today (Buzsáki et al., 2013). Despite the increase in human brain volume, the frequency hierarchy that characterizes neural oscillations in the brain and body has been largely conserved across species. Animal cerebral oscillatory interaction is assumed to be equally causal as appears to be in humans. Animal studies are often conducted as suitable stand-ins for the usually invasive testing procedures and the findings extended to explain human oscillatory dynamics (Roelfsema et al., 1997; Tallon-Baudry et al., 2004; Paulk et al., 2013). This section will review an integrative example of similar extracerebral shared resonance to support additional research into non-human brain-body oscillatory links.

Tort et al. (2018) examined nasal respiratory entrained rhythms in rodents, in a system similar to the speculated olfactory-brain shared resonance in humans. Rodent respiration cycles can occur at frequencies as low as 1 Hz during rest/sleep and as high as 14 Hz during sniffing, making these rhythms indistinguishable from delta and theta oscillations in the rodent brain if not measured concomitantly (Wesson et al., 2008; Rojas-Líbano et al., 2014). When differentiating the respiration rhythms from low frequency brain waves, respiration-gamma coupling is observable in several regions of the brain. During immobility and active behaviors, the respiration-gamma coupling is recorded in the olfactory bulb (OB), medial prefrontal cortex (mPFC), and hippocampus. This coupling ceases if the rodent diverts breathing to the trachea or the OB is surgically removed. The mechanism is unknown, but Tort hypothesized the respiration-gamma coupling serves as a means of integrating gamma activity across distant regions given that slow oscillations, such as that of the respiratory cycles, remain coherent while traveling longer spatial distances.

Tsukahara et al. (1973), in observing neural oscillations from an anesthetized *O. vulgaris* retina, serendipitously recorded retinal oscillations resonating with respiration. Spontaneous oscillations appeared as bursts at approximately 0.2 Hz during the measurement of retinal oscillations under illumination. The spontaneous bursts ceased when the octopus breathing was interrupted. Similar in kind to speculated olfactory shared resonance, the neural oscillations commenced at inspiration and somewhat ceased at expiration. This is likely evidence for a shared resonance relationship between the octopus’s neural and physiological rhythms.

Shared resonance between the central and peripheral nervous system, as well as in other organs, is likely not isolated to humans. Beginning with physiological entrainment of neural rhythms, a similar frequency hierarchy of resonating brain-body oscillations presumably awaits discovery in other animal species.

## Discussion

Taken together, our developing model supposes functional links between endogenous neural rhythms throughout the brain and body. The peripheral nervous clusters in the organs can be regarded in some manner as extensions of the brain itself, and the consciousness associated with the activity of the cerebral brain can likewise be extended to encompass the entire organism, although in a more rudimentary manner. This is exemplified in the gastric influence on interoception, the heart on emotional processing, and the rhythms of the eye and retina in active visual sensing. This is supplemented by corticospinal shared resonance in locomotion and respiration-gamma coupling in emotional processes. The similarities with animal neural-physiological synchronization indicates a probable universality of the oscillatory substrates of consciousness. Theories of embodied cognition are congruent; the timeline of development suggests all modern organisms are likely to exhibit some manner of brain-body coupling and the included discussion on respiration-neural coupling in rats and octopi would support it.

The spatiotemporal hierarchy depicted in Table 1 generally supports GRT’s predictions and closely aligns with Klimesch’s (2018) similar binary hierarchy brain body oscillation theory shown in Figure 1. Data supports the suggested inverse relationship between resonating frequencies and the spatial distance separating the resonating clusters.

For clinical applications this model presents a novel path by which the documentation and treatment of neuropsychiatric conditions may be extrapolated to the whole organism. Findings of bodily illness coinciding with cognitive conditions are well documented and, in some cases, bi-directional. A pathological reduction of HRV is associated with PTSD (Whitehouse and Heller, 2008), Alzheimer’s Disease (Zulli et al., 2005), and anxiety disorders (Chalmers et al., 2014). Trauma disorders are frequently comorbid with functional gastro-intestinal disorders (Kolacz et al., 2019) and chronic gut inflammation is linked to the development and progression of AD (Santiago and Potashkin, 2021). To assume disruption of the underlying synchrony architecture that may influence the trajectory and symptomatology of a disorder in such cases is not so great a leap of faith. In fact, Richter et al.’s (2017) findings of gastric-alpha coupling prompted some authors to consider this synchrony link as a viable route by which the gut microbiota, implicated in a number of disorders (Rees, 2014; Latalova et al., 2017; Sochocka et al., 2019), may be able to influence cognitive processes (Palacios-García and Parada, 2020). The benefits of this model as compared to previous efforts is the quantifiable nature by which the strength of brain-body coupling may be examined through the use of already developed phase-to-phase and phase-amplitude quantification techniques. With proper development, the spatiotemporal hierarchy of brain-body shared resonance may be utilized in illustrating a more comprehensive picture of holistic health, and to provide the means by which treatments based on correcting out of sync brain and body rhythms may be administered as an effective adjunct to prevailing treatments.

Subsequent efforts should be directed at further developing the hierarchy of brain-body neural resonances. Başar and Klimesch’s foundational works suggest coupling between the brain and all of the body’s organs. Corticospinal and olfactory-brain shared resonance relationships require further development to integrate into the model. A related venture is the examination of environmental EM influence, such as geomagnetic activity (Bureau and Persinger, 1992; Cherry, 2002, 2003) or anthropogenic sources more generally (Becker et al., 1985), upon this network of resonating structures.

## Author Contributions

AY produced the manuscript. TH and ME advised and edited. All authors contributed to the article and approved the submitted version.

## Conflict of Interest

The authors declare that the research was conducted in the absence of any commercial or financial relationships that could be construed as a potential conflict of interest.

## Publisher’s Note

All claims expressed in this article are solely those of the authors and do not necessarily represent those of their affiliated organizations, or those of the publisher, the editors and the reviewers. Any product that may be evaluated in this article, or claim that may be made by its manufacturer, is not guaranteed or endorsed by the publisher.
